# Association between Serum Selenium Concentrations and Levels of Proinflammatory and Profibrotic Cytokines—Interleukin-6 and Growth Differentiation Factor-15, in Patients with Alcoholic Liver Cirrhosis

**DOI:** 10.3390/ijerph14040437

**Published:** 2017-04-21

**Authors:** Andrzej Prystupa, Paweł Kiciński, Dorota Luchowska-Kocot, Anna Błażewicz, Jarosław Niedziałek, Grzegorz Mizerski, Mariusz Jojczuk, Andrzej Ochal, Jarosław J. Sak, Wojciech Załuska

**Affiliations:** 1 Department of Internal Medicine, Medical University of Lublin, Staszica 16, 20-081 Lublin, Poland; aprystup@wp.pl; 2 Department of Family Medicine, Medical University of Lublin, Staszica 11, 20-081 Lublin, Poland; pawelkici@wp.pl (P.K.); grzegorz.mizerski@umlub.pl (G.M.); 3 Department of Medical Chemistry, Medical University of Lublin, Chodźki 4a (Collegium Pharmaceuticum), 20-093 Lublin, Poland; dorota.luchowska-kocot@umlub.pl; 4 Department of Analytical Chemistry, Medical University of Lublin, Chodźki 4a (Collegium Pharmaceuticum), 20-093 Lublin, Poland; anna.blazewicz@umlub.pl; 5 Individual Medical Practice, Lublin, Ludwika Hirszfelda 5/11, 20-092 Lublin, Poland; niedzialek.jarek@poczta.fm; 6 Department of Trauma Surgery and Emergency Medicine, Medical University of Lublin, Staszica 16, 20-081 Lublin, Poland; mariusz.jojczuk@umlub.pl (M.J.); andrzej.ochal@umlub.pl (A.O.); 7 Department of Ethics and Human Philosophy, Medical University of Lublin, Staszica 4/6 (Collegium Maximum), 20-059 Lublin, Poland; 8 Department of Nephrology, Medical University of Lublin, Jaczewskiego 8, 20-954 Lublin, Poland; wtzaluska2@poczta.onet.pl

**Keywords:** alcohol, liver cirrhosis, selenium, interleukin-6, growth differentiation factor-15

## Abstract

According to some authors, serum selenium levels are strongly associated with the severity of liver diseases, including liver cirrhosis. The aim of this study was to determine the relationship between the concentration of selenium and pro-inflammatory and profibrotic cytokines—interleukin-6 (IL-6) and growth differentiation factor 15 (GDF-15) in patients with alcoholic liver cirrhosis. The parameters studied were determined in the serum of 99 patients with alcoholic liver cirrhosis divided based on the severity of disease according to the Child-Turcotte-Pugh criteria. In patients with liver cirrhosis, the serum selenium concentration was statistically lower, whereas serum IL-6 and GDF-15 concentrations were higher than those in the control group. Moreover, the concentration of selenium negatively correlated with the levels of GDF-15 and IL-6. The above results may indicate a role of selenium deficiency in the pathogenesis and progression of alcoholic liver disease.

## 1. Introduction

Alcohol remains a major cause of liver disease worldwide. Alcohol liver cirrhosis includes medical conditions ranging from simple steatosis to decompensated cirrhosis [1] and is the twelfth most frequent cause of death in the United States, with an age–adjusted rate of 9.6 per 100,000 population [2]. The precise molecular pathways of the initiation and progression of alcohol-induced liver tissue injury are not fully understood, but it has been well established that alcohol toxicity to organs is connected with the generation of oxidative and non-oxidative ethanol metabolites and the translocation of gut-derived endotoxins into the bloodstream. These processes lead to cellular injury and stimulation of inflammatory responses releasing a great variety of cytokines. Continuation of alcohol abuse progresses the injury through impairment of tissue regeneration and extracellular matrix turnover, leading eventually to fibrogenesis and cirrhosis [3].

Selenium, an essential trace mineral for humans, is delivered to the body mainly via food and water. Selenium has antioxidant properties and is a cofactor of many enzymes, including glutathione peroxidase. Currently, this element is considered to be an anticancer agent that prevents the processes of cell proliferation and tumor growth [4]. According to some authors, the serum selenium level is strongly associated with the severity of liver damage [5,6]. However, it should be emphasized that both the excess and deficiency of selenium exert adverse effects. The aim of this study was to determine the relationships between the concentration of selenium and pro-inflammatory and profibrotic cytokines—interleukin-6 (IL-6) and growth differentiation factor 15 (GDF-15)—in patients with alcoholic liver cirrhosis.

## 2. Experimental Section

### 2.1. Patients

The study included 99 patients with diagnosed alcoholic cirrhosis from the region of Lublin, (Eastern Poland). All patients enrolled in the study had consumed large amounts of alcohol for a long time. The assessment of alcohol consumption was based on a self-reported-survey. The mean duration of alcohol abuse in cirrhotic patients was 14.4 ± 5.1 years and mean quantity of alcohol consumed was 5.1 ± 2.8 drinks in men and 4.5 ± 2.6 drinks per day in women (one drink = 10 g of alcohol). Subjects with viral and autoimmune diseases were excluded from the study. Liver cirrhosis was diagnosed based on clinical features, history of heavy alcohol consumption, laboratory tests and abdominal ultrasonography. The patients with alcoholic hepatitis were excluded. The degree of liver cirrhosis was evaluated according to the Child-Turcotte-Pugh criteria (Child-Pugh score) [7]. Based on these the patients were assigned to one of three groups: 1st-P-Ch A-29 with stage A, 2nd-P-Ch B-26 with stage B and 3rd-P-Ch C-34 with stage C of liver cirrhosis. The control group consisted of 20 healthy individuals without liver disease who did not abuse alcohol. None of the patients or healthy participants received mineral supplements. Both clinical assessment and laboratory tests were used to exclude the underlying liver diseases in the control group. There were no significant age- or gender-related differences in the subgroups (Table 1). Detailed demographic, clinical and biochemical characteristics of the patients are presented in Table 1 and Table 2. The study protocol was approved by the Bioethics Committee at the Medical University of Lublin, Poland (agreement number KE-0254/349/2015). All subjects gave their written informed consent for participation in the study.

### 2.2. Human Serum Samples

The studied material consisted of 119 human serum samples. These samples were taken from healthy individuals and patients with alcoholic liver cirrhosis and analyzed in the same way. They were transported and stored in polypropylene containers. The digestion was carried out in the *Nova*WAVE Microwave Tunnel Digestion System (SCP Science, Montreal, QC, Canada) using Teflon^®^ vessels (SCP Science, Montreal, QC, Canada). The microwave-assisted sample preparation was conducted in a closed system. Each time an acidic digestion with 65% nitric acid water solution was applied (1 mL of HNO_3_: 9 mL of deionized H_2_O). The optimized conditions of the mineralization procedure had been previously published [8]. The obtained solutions were poured into volumetric flasks (PTFE) and diluted with deionized water (18 MΩ·cm) whenever necessary before final analysis.

### 2.3. Instrumentation and Reagents

After mineralization, each human serum sample (1 mL) was analyzed at least in triplicate using a high-resolution atomic absorption spectrometer. The measurements were performed with the ContrAA700 high-resolution continuum source graphite tube AAS instrument (Analytik Jena AG, Jena, Germany). A transversely heated graphite furnace was used as an atomizer. The parameters were set as follows: wavelengths = 196.0267 nm, pyrolysis temperature = 950 °C, atomization temperature = 1900 °C, and 5 µL Pd(NO_3_)_2_ (0.1% Pd) as a modifier. The concentration of a stock solution (100 µg/L Se in 1% HNO_3_) was prepared.

The method accuracy was verified by the use of Seronorm™ Trace Elements Serum L-2 (Billingstad, Norway) human serum certified reference material. The average recovery for five separate determinations was 97.8%. The limit of detection (3σ) was estimated to be 2.00 µg/L. All reagents used were of at least analytical grade. Water with a resistivity of 18.2 MΩ·cm was deionized in a Milli-Q system (Millipore, Bedford, MA, USA); 65% nitric acid solution and other stock solutions were purchased from Sigma-Aldrich (Darmstatd, Germany).

The serum interleukin-6 concentration was determined using the Human Interleukin-6 ELISA Kit (BioVendor, Brno, Czech Republic) according to the manufacturer’s procedure, i.e., sandwich immunoassay where an anti-Human IL-6 coating antibody is absorbed onto microwells. After the addition of an appropriately diluted sample or a standard, human IL-6 bound to antibodies was absorbed to the microwell. Subsequently, a biotin-conjugated anti-Human IL-6 antibody was added and bound to human IL-6. Unbound biotin-conjugated anti-Human IL-6 antibody was removed and streptavidin-HRP was added and bound to the biotin conjugated anti-Human IL-6 antibody. During the next step, unbound streptavidin-HRP was removed and a substrate solution reactive with HRP was added to the wells. A colored product was formed in proportion to the amount of human IL-6 and its concentration was determined using the Epoch Microplate Spectrophotometer (BioTek Instrumentals, Inc., Winooski, VT, USA).

The serum growth differentiation factor 15 was determined applying Human GDF-15 ELISA sandwich enzyme immunoassay (BioVendor) for the quantitative measurement of human GDF-15/MIC-1 (growth differentiation factor 15/macrophage inhibitory cytokine 1). The standards and samples were incubated in microtitrate wells pre-coated with polyclonal anti-human GDF-15 antibody. After incubation, biotin labeled polyclonal anti-human GDF-15 was added. The further procedure (addition of streptavidin-conjugated to (horseradish peroxidase, HRP) and substrate solution, etc.) was analogous to the human IL-6 assays.

### 2.4. Statistical Analysis

STATISTICA 12 PL (StatSoft, Inc., Tulsa, OK, USA) was used for data analysis. Continuous variables were expressed as mean ± standard deviation (SD). Before calculations, variables were checked for normality using the Shapiro-Wilk test; the Brown-Forsythe test was applied to test equality of variances. To compare continuous variables between two groups (the control and the study group), the Mann-Whitney test was used; for more than two groups, the Kruskal-Wallis rank test was used, a nonparametric equivalent of ANOVA. The Dunn test was applied for detailed identification of statistically different groups. Correlations among variables were tested using Spearman’s rank correlation. Qualitative variables were shown as indicators of structures and compared using the χ^2^ test. For all tests, *p* < 0.05 was considered as statistically significant.

## 3. Results

### 3.1. Serum Selenium Concentration in Patients with Alcoholic Liver Cirrhosis Compared to the Control Group

**Figure 1:**
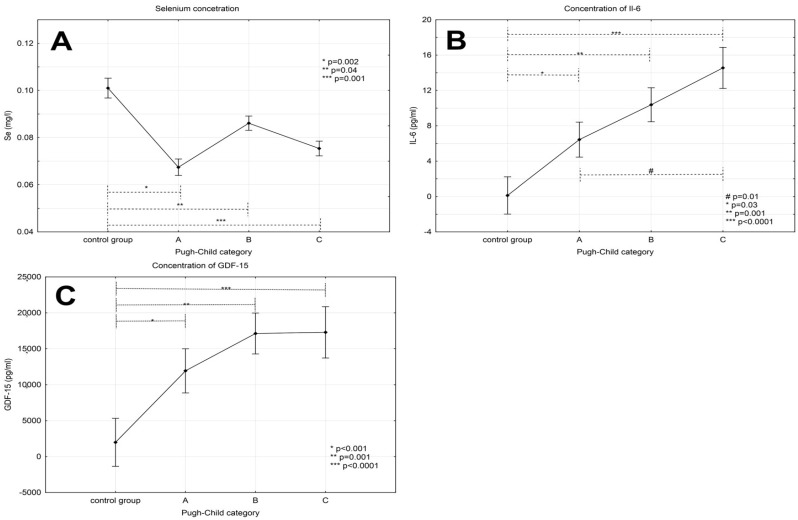
Concentrations of selenium (**A**), IL-6 (**B**) and GDF-15 (**C**) in patients with alcoholic liver cirrhosis and in the control group. Values expressed as the mean ± standard deviation.

The serum selenium concentration was lower in patients with liver cirrhosis than in the control group (0.1 ± 0.013 mg/L); its values were 0.068 ± 0.014 mg/L in stage A of disease, 0.085 ± 0.016 mg/L in stage B and 0.072 ± 0.017 mg/L in stage C (Figure 1A) according to the Child-Pugh criteria.

Post-hoc tests revealed significant differences in concentrations of this element between the control group and groups with various stages of liver cirrhosis (*p* values were respectively 0.002, 0.04 and 0.001 for the control group vs. A, B and C Child-Pugh). However, no statistically significant differences in the concentrations of Se were demonstrated comparing particular groups of liver cirrhosis.

### 3.2. Levels of Interleukin-6 in Patients with Alcoholic Liver Cirrhosis

The highest concentration of interleukin-6 was observed in patients with decompensated cirrhosis (14.55 ± 12.51 pg/mL in stage C and 10.38 ± 9.96 pg/mL in stage B) whereas the lowest one in the control group (0.12 ± 0.41 pg/mL). Multiple comparison tests revealed significant differences between the control group as compared to the groups with various stages of severity of liver cirrhosis—A, B and C (*p*-value: 0.03, 0.001 and <0.0001, respectively). In addition, a statistically significant difference between patients with stage A and C according to Child-Pugh was observed (*p* = 0.01) (see Figure 1B).

### 3.3. Levels of GDF-15 in Patients with Alcoholic Liver Cirrhosis

The highest concentration of GDF-15 (17.86 ± 7.2) was noticed in stage C patients. Moreover, its concentration decreased in less advanced stages of disease (stage B—16.53 ± 8.01 and stage A—12.49 ± 7.93). The lowest value of GDF-15 concentration was observed in the control group (1.98 ± 0.88). Multiple comparison tests demonstrated significant differences in the levels of this parameter comparing the control group versus groups with various stages of disease severity (*p*-values for comparisons with phases A, B and C were <0.001; 0.001 and <0.0001, respectively). However, there were no significant intergroup differences in the concentrations of GDF-15 in patients with different stages of cirrhosis (see Figure 1C).

### 3.4. Correlations among Serum Selenium Concentration and GDF-15 and IL-6

The concentration of selenium negatively correlated with the concentration of GDF-15 (*r* = −0.29; *p* = 0.03) and the concentration of interleukin-6 (*r* = −0.38; *p* = 0.03), but not with the CRP level. In addition, there was a positive correlation between the concentration of GDF-15 and interleukin-6 (*r* = 0.64; *p* < 0.0001) (Table 3). However, there were no significant correlations between serum interleukin-1 or GDF-15 and gender, age as well as duration of alcohol abuse.

Moreover, there was no correlation between age and the concentration of GDF-15 (*p* = 0.46) as well as of Il-6 (*p* = 0.27). Otherwise, a weak negative correlation was observed between age and the concentration of selenium (*r* = −0.26; *p* = 0.01). Furthermore, no significant differences were found in the concentrations of GDF-15 (*p* = 0.87), Il-6 (*p* = 0.3) and selenium (*p* = 0.63) between male and female patients.

## 4. Discussion

Cirrhosis, the end-stage of alcoholic liver disease, is associated with many metabolic disorders, including abnormal metabolism of trace elements [9]. Our results indicate a selenium deficiency in patients with liver cirrhosis and are consistent with previously published study findings. Nangliya et al. showed the relationship between the severity of liver cirrhosis and reduced concentration of selenium [10]. Kazi et al. noticed that the concentrations of selenium and zinc were reduced in patients with cirrhosis and liver cancer and negatively correlated with the concentrations of heavy metals (cadmium and arsenic) [11]. According to Martinez-Peinado et al., the significantly reduced concentration of selenium in patients with cirrhosis did not correlate with the disease severity evaluated by Child-Pugh criteria [12]. In the same study, a positive correlation between the selenium concentration and age of patients was demonstrated. Interestingly, the levels of this element in men were higher in comparison to women. In our study, the concentration of selenium in patients with cirrhosis was significantly lower compared to healthy individuals, but the differences among various Child-Pugh stages of disease were not statistically significant. Moreover, no association was found between serum selenium and age, sex of patients or duration of alcohol addiction. We have studied a homogeneous group consisted of patients exclusively with alcoholic liver cirrhosis. On the contrary, individuals with different causes of liver diseases and with hepatocellular carcinoma were included in the cited above papers. This can potentially affect the reported discrepancies in the results.

Proinflammatory cytokines, such as interleukin-1 and -6 or tumor necrosis factor α (TNF-α), are involved in the pathogenesis of hepatic cirrhosis. They are produced by the Kupffer cells and play a role in sustaining the inflammatory process associated with fibrosis, intensification of necrosis and apoptosis of hepatocytes [13,14]. The reduced concentration of selenium in patients with liver cirrhosis may be related to the pathophysiological processes corresponding to the progression of disease. Himoto et al. found that low concentrations of selenium in patients with hepatitis C-related cirrhosis was associated with increased insulin resistance and negatively correlated with the severity of fibrosis [15].

Several literature reports stressed the hepatoprotective effect of selenium. In a rat model, the administration of probiotics containing selenium was demonstrated to inhibit liver fibrosis induced by carbon tetrachloride, which, according to the authors, was likely to be associated with reduced oxidative stress, inflammation and stellate cells apoptosis [16,17]. Furthermore, a hepatoprotective impact of selenium on toxic thioacetamide-induced liver injury was reported in a rat model [18]. In another study, selenium supplementation was found to decrease liver fibrosis by inhibiting the expression of NFκB and TGF-β [19]. Mertens et al., who studied cell cultures, showed that under conditions mimicking sepsis, decreased levels of zinc and selenium were associated with intensified oxidative stress and elevated levels of interleukin-6 [20]. Pei et al. demonstrated that sodium selenite inhibited lipopolysaccharide (LPS) induced expression of VEGF, TGF beta, and interleukin-6 in cultured human cells PC3 [21]. Moreover, according to Shilo et al., selenium supplementation was associated with increased hepatic expression of manganese superoxide dismutase—a key antioxidant enzyme—as well as decreased production of IL-6 by Kupffer cells in animals treated with LPS [22].

The available experimental evidence indicates a protective effect of selenium on the liver resulting primarily from the inhibition of fibrosis and oxidative stress. Some studies focused on selenium supplementation in patients with liver cirrhosis. Burk et al. observed that not all forms of supplementation were equally effective in patients with liver cirrhosis and selenium deficiency. The authors reported considerably better results after selenate supplementation, as compared to selenomethionine [23].

The findings presented by Tseng et al. are of interest. In their study, the concentration of interleukin-6 negatively correlated with the concentration of selenium in the group of elderly patients [24]. Not all the studies published, however, have provided consistent results. Ansar et al. demonstrated that exposure of rats to acute (not chronic) toxic effects of mercury chloride, and, in addition, selenium supplementation, resulted in higher concentrations of TNF, IL-6 and IL-10 [25]. Furthermore, Daeian et al. did not show the influence of selenium supplementation on the concentration of proinflammatory cytokines such as TNF, IL-1 beta and IL-6 in patients after autologous stem cell transplantation [26]. In our research, the concentration of selenium was negatively correlated with the level of Il-6 what indicates the negative significance of the deficiency of this element in cirrhotic patients. However, the relationship between selenium and this proinflammatory cytokine may vary depending on the type of pathological condition and its duration (acute or chronic).

GDF-15 is a cytokine involved in the process of fibrosis and the pathogenesis of numerous diseases including liver cirrhosis. Liu et al. demonstrated increased expression of GDF-15 in patients with cirrhosis and HCV-related hepatocellular carcinoma in comparison to healthy subjects. In asymptomatic carriers of HCV and HBV, concentrations of GDF-15 are intermediate (lower than in cancer and cirrhosis patients and higher than in healthy individuals) [27]. In the study by Lee et al., the concentration of GDF-15 correlated with the severity of disease (the highest concentration was observed in patients with decompensated cirrhosis, lower in the case of compensated cirrhosis and chronic hepatitis). However, in their study, patients with alcoholic liver disease constituted only a quarter of the study population; the other patients suffered from hepatitis or autoimmune liver disease of unknown etiology [28]. In our study, elevated levels of GDF-15 were observed in patients with alcoholic cirrhosis compared to healthy subjects. Moreover, the concentration of this cytokine in patients with decompensated cirrhosis (stages B and C according to Child-Pugh scores) was higher than that in stage A patients, yet the differences were not statistically significant. To our best knowledge, our study findings are the first to demonstrate a negative correlation between the concentration of selenium and profibrotic and proinflammatory cytokines, i.e., IL-6 and GDF-15, in patients with alcoholic liver cirrhosis.

## 5. Conclusions

In our study, decreased serum selenium concentrations and a negative correlation between serum Se and concentrations of interleukin-6 and GDF-15 were noticed in patients with alcoholic liver cirrhosis, which may indicate a role of selenium deficiency in the pathogenesis and progression of alcoholic liver disease.

## Figures and Tables

**Table 1:** Demographic and clinical characteristics of the study and control groups (mean ± SD).

Characteristic	Control Group (*n* = 20)	Alcoholic Liver Cirrhosis (*n* = 99)		
		P-Ch A (*n* = 29)	P-Ch B (*n* = 36)	P-Ch C (*n* = 34)
Age (years)	48.9 ± 15.1	53.3 ± 12.3	54.6 ± 11.0	56.9 ± 7.7
The percentage of men	65%	72.4%	66.7%	58.8%
Height (cm)	170.4 ± 6.6	171.8 ± 7.8	175.3 ± 8.4	173.4 ± 6.9
Body mass (kg)	67.8 ± 7.8	68.1 ± 14.8	71.5 ± 13.2	70.2 ± 12.8
Time of alcohol abuse (years)	-	13.1 ± 4.8	14.1 ± 4.9	15.7 ± 5.4
Ascites (%)	0	32%	61%	85%
Esophageal varices (%)	0	14%	45%	88%
Encephalopathy (%)	0	28%	51%	91%

**Table 2:** Biochemical characteristics of the study and control groups (mean ± SD).

Caracteristic	Control Group (*n* = 20)	Alcoholic Liver Cirrhosis (*n* = 99)		
		P-Ch A (*n* = 29)	P-Ch B (*n* = 36)	P-Ch C (*n* = 34)
Bilirubin (mg/dL)	0.68 ± 0.28	2.21 ± 1.4	4.12 ± 3.25	7.89 ± 7.94
Albumin (g/dL)	-	3.27 ± 0.76	2.78 ± 0.6	2.44 ± 0.46
INR	-	1.25 ± 0.27	1.44 ± 0.29	1.68 ± 0.41
Blood platelets (g/L)	226.8 ± 35.8	186.03 ± 76.9	123.6 ± 66.25	114.11 ± 61.6
Mean cell volume (fL)	94.65 ± 4.45	92.38 ± 6.25	91.9 ± 10.08	97.53 ± 8.02
Urea (mg/dL)	-	32.04 ± 20.1	23.49 ± 15.62	39.58 ± 16.1
Sodium (mmol/L)	139.82 ± 3.24	133.67 ± 5.3	135.38 ± 3.6	133.51 ± 6.63
Potassium (mmol/L)	4.41 ± 0.37	3.88 ± 0.6	3.94 ± 0.6	3.3 ± 0.66
C-reactive protein (mg/L)	2.11 ± 1.96	14.97 ± 12.62	19.21 ± 17.35	20.8 ± 19.92

**Table 3:** Correlation between serum concentrations of selenium and growth differentiation factor-15, interleukin-6 and C-reactive protein.

Variable		Correlation Coefficient *p*-Value
Selenium	GDF-15	*r* = −0.29
		*p* = 0.03
	IL-6	*r* = −0.38
		*p* = 0.03
	CRP	*r* = −0.17
		NS

NS—not significant.
